# RBL2/p130: a direct AKT substrate and mediator of AKT inhibition-induced apoptosis

**DOI:** 10.18632/oncoscience.467

**Published:** 2018-08-28

**Authors:** Elisa Ventura, Francesca Pentimalli, Antonio Giordano

**Affiliations:** Sbarro Institute for Cancer Research and Molecular Medicine, Center for Biotechnology, College of Science and Technology, Temple University, Philadelphia, PA 19122, USA; Department of Medicine, Surgery and Neuroscience, University of Siena, 53100, Siena, Italy

**Keywords:** RBL2/p130, AKT, apoptosis, lung cancer, mesothelioma

Retinoblastoma-like (RBL)2/p130, along with the other two retinoblastoma (RB)1/p105 and RBL1/p107 pocket proteins, forms the RB protein family. RB proteins, whose activity is deregulated in the vast majority of human tumors, are oncosuppressor molecules and play a key role in cell cycle regulation, in cellular differentiation, senescence and apoptosis [1]. RB proteins control gene expression by binding to the E2F transcription factors and by recruiting chromatin- and histone-modifying enzymes. RB protein functions are modulated by their phosphorylation status [1]. Cyclin-dependent kinases (CDKs) are the main kinases responsible for RBL2/p130 phosphorylation, however different non-CDK phosphorylation sites have been identified in RBL2/p130 and, in G0 arrested cells, RBL2/p130 has been recognized as a glycogen synthase kinase (GSK) 3 substrate as well [2]. Following up a study in which interfering with the CDK2-mediated inhibitory phosphorylation of RBL2/p130 induced apoptosis in various cancer cell lines (Pentimalli F, et al. Cancer Res. 2015; 75:LB-080), Pentimalli et al. demonstrated that RBL2/p130 is a direct substrate of AKT, a key antiapoptotic protein driving cancer cell proliferation [3]. The Authors showed that RBL2/p130 and AKT1 physically interact and that AKT1 phosphorylates the previously unreported residue Ser941, within the pocket domain. Notably, in lung cancer and mesothelioma cells, both characterized by high AKT activity, AKT inhibition prevented Ser941 RBL2/p130 phosphorylation and was associated with increased RBL2/p130 levels and stability, suggesting a role for AKT in inhibiting RBL2/p130 function [3]. These findings further challenge the canonical model of RB proteins being inactivated principally by CDKs and suggest that kinases other than CDKs may directly control RB proteins activity/stability. Further studies will shed light on the molecular mechanisms underlying the regulation of RBL2/p130 activity/stability by AKT, i.e. whether AKT-dependent RBL2/p130 phosphorylation modulates RBL2/p130 nucleo-cytoplasmic shuttling, degradation, phosphorylation by CDKs or interaction with other molecules such as the E2F transcription factors or the other components of the dimerization-partner, RB-like, E2F and multi-vulval class B (DREAM) complex, a key regulator of quiescence to which both RB1/p105 and RBL1/p107 belong. Further studies will also disclose whether the other two members of the RB family, RB1/p105 and RBL1/p107, are AKT substrates too or whether a direct regulation by AKT is peculiar of RBL2/p130, distinguishing RBL2/p130 from the other two pocket proteins and in particular from the closely related RBL1/p107.

**Figure 1:**
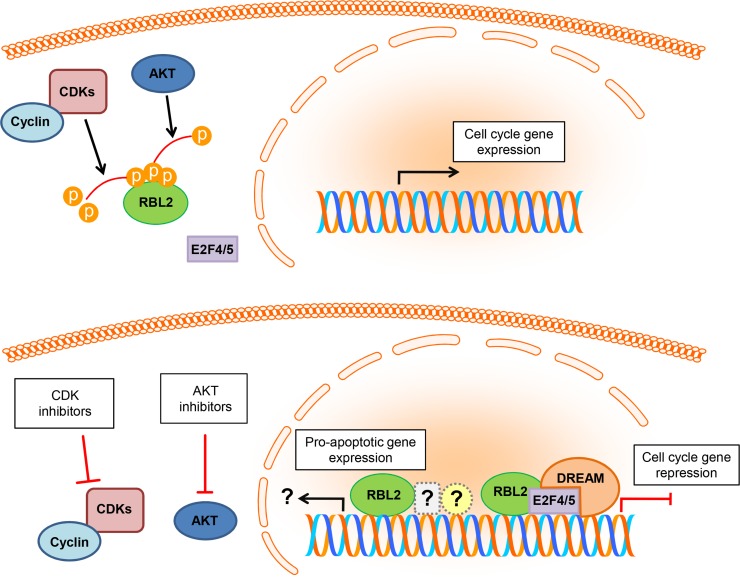
Schematic representation of the hypothesized model of RBL2/p130 regulation by cyclin-dependent kinases (CDKs) and AKT RBL2/p130 phosphorylation by CDKs and AKT leads to RBL2/p130 inactivation and to the consequent activation of cell cycle genes (upper illustration). The combined inhibition of CDKs and AKT would synergize in promoting RBL2/p130 activation and nuclear accumulation, with the consequent inhibition of the expression of cell cycle genes, through E2F4/5 binding and likely the DREAM complex, and possible activation of pro-apoptotic genes, through unknown partners (lower illustration).

Interestingly, the increase in RBL2/p130 levels and stability triggered by AKT inhibition was paralleled by an increase in the levels and stability of the CDK inhibitor p27, by G0/G1 cell cycle arrest and by caspase-dependent apoptosis [3]. The role played by RBL2/p130 in apoptosis is a matter of debate since RBL2/p130 can be both pro- and anti-apoptotic, depending on the cellular context [4]. The data reported by Pentimalli et al. suggest that in tumor cells characterized by AKT over-activation, AKT prevents apoptosis at least in part by affecting RBL2/p130. Indeed, AKT inhibition translated into the nuclear accumulation of RBL2/p130 which, in turn, promoted the expression of the pro-apoptotic gene *TP73* [3]. Interestingly, the apoptosis induced upon AKT inhibition was reduced in different cell lines silenced for RBL2/p130. These data broaden the current knowledge on the molecular mechanisms behind AKT anti-apoptotic role, identifying in RBL2/p130 a key mediator of this process (Figure 1).

The AKT signaling pathway is deregulated in many tumor entities. Therefore, as a key regulator of several cellular processes including cell survival, cell growth, cell proliferation and glucose homeostasis and being hyperactivated in more than 50% human tumors where it promotes tumor growth and progression, AKT represents an attractive therapeutic target [5]. Several AKT inhibitors have been generated and are currently in clinical development [5]. Similarly, as RB proteins are deregulated in the majority of human tumors, different strategies aiming at re-establishing RB protein function have been proposed as a therapeutic approach for the treatment of cancer patients [4]. These strategies mainly rely on the use of CDKs inhibitors, which have recently entered the clinical practice with CDK4/CDK6 inhibitors being approved for the treatment of advanced/metastatic breast cancer [6]. Notably, Pentimalli and coworkers could demonstrate that the combined used of the pan-CDK inhibitor roscovitine, potently active against CDK2, and the AKT inhibitor VIII (AKTiVIII) was synergic in lung cancer and mesothelioma cells [3]. Considering the therapy-resistance of these tumors and the urgent need for alternative treatment options [7], these results are of particular interest for their translational potential. Further *in vivo* studies will elucidate the potential synergistic anti-tumoral therapeutic efficacy of the combined use of CDK and AKT inhibitors. Also, since the loss of RBL2/p130 expression and the hyperactivation of AKT signaling are common traits of many tumor entities, these findings may be potentially extended to other tumor types.
